# Unraveling the intricate link between cell death and neuroinflammation using *Drosophila* as a model

**DOI:** 10.3389/fcell.2024.1479864

**Published:** 2024-10-01

**Authors:** Pooja Rai, Andreas Bergmann

**Affiliations:** Department of Molecular, Cell and Cancer Biology, UMass Chan Medical School, Worcester, MA, United States

**Keywords:** antimicrobial peptides (AMPs), dopaminergic neurons (DNs), Toll pathway, neuroinflammation, *Drosophila*, alpha (α)-Synuclein

## Abstract

Protein aggregation is a common pathological occurrence in neurodegenerative diseases. This often leads to neuroinflammation, which exacerbates the aggregation and progression of diseases like Parkinson’s and Alzheimer’s. Here, we focus on immune responses and neurotoxicity in a Parkinson’s disease model in *Drosophila*. Mutations in the SNCA gene that encodes the alpha (α)-Synuclein protein have been linked to familial Parkinson’s disease, disrupting autophagy regulation in neuronal cells and promoting the formation of Lewy bodies, a hallmark of Parkinson’s pathology. This results in the loss of dopaminergic neurons, manifesting as movement disorders. α-Synuclein aggregation triggers innate immune responses by activating microglial cells, leading to phagocytic activity and the expression of neuroprotective antimicrobial peptides (AMPs). However, sustained AMP expression or chronic inflammation resulting from inadequate microglial phagocytosis can induce neuronal toxicity and apoptosis, leading to severe dopaminergic neuron loss. This review underscores the mechanistic connection between immune response pathways and α-Synuclein-mediated neurodegeneration using *Drosophila* models. Furthermore, we extensively explore factors influencing neuroinflammation and key immune signaling pathways implicated in neurodegenerative diseases, particularly Parkinson’s disease. Given the limited success of traditional treatments, recent research has focused on therapies targeting inflammatory signaling pathways. Some of these approaches have shown promising results in animal models and clinical trials. We provide an overview of current therapeutic strategies showing potential in treating neurodegenerative diseases, offering new avenues for future research and treatment development.

## 1 Background

Understanding the intricacies of neuronal cell death has long been a challenging pursuit in the realm of neuroscience. Over decades of meticulous molecular dissection, researchers have unraveled the mysteries surrounding this phenomenon. It is now widely recognized that, in most instances, neuronal cell death is not merely a random occurrence but rather the culmination of well-coordinated programs initiated by the neuron itself in response to various internal or external signals throughout its lifespan. During the developmental stages of the human central nervous system (CNS), neurogenesis is often accompanied by substantial neuronal cell loss (Okano and Sawamoto, 2008; Paridaen and Huttner, 2014; Chi et al., 2018). This process is integral to constructing a fully functional command center. However, in mature CNSs, extensive neuronal loss is a rare occurrence, with occasional or planned death events. Nonetheless, observable differences in neuron numbers between young and old individuals may emerge in certain brain regions during the aging process (Grady, 2012; Fjell et al., 2010). While limited neuronal loss characterizes normal aging, neurodegenerative diseases present a stark departure from this pattern. These conditions are marked by a significant increase in neuronal loss compared to age-matched controls, a trend that correlates closely with longitudinal examinations of disease progression. The clinical implications of these observations are immense, fueling intense interest in deciphering the triggers of pathological changes leading to cell death and regional brain shrinkage. Such insights hold the promise of guiding the development of treatments aimed at halting or reversing disease progression. In general, mature CNS neurons exhibit remarkable resistance to cell death compared to their immature counterparts. These neurons, having endured an individual’s lifetime, possess sophisticated mechanisms for maintaining cellular homeostasis and resilience to various stresses. Nonetheless, cell death becomes an inevitable outcome when the cumulative burden of stressors overwhelms the neuron’s capacity for resilience—a scenario commonly observed in neurodegenerative diseases (Lau et al., 2023). Thus, unraveling the mechanisms underlying neuronal resilience and vulnerability holds the key to understanding and combating these devastating conditions.

Ensuring the proper functioning of cells and organisms hinges on the correct activity of a vast network of proteins. Central to this functionality is the three-dimensional structure of proteins, dictated by their amino acid sequences. Chaperone proteins play a pivotal role in overseeing protein folding processes, minimizing errors, and facilitating the removal of malfunctioning proteins. However, mounting evidence suggests that protein misfolding and aggregation underlie various neurological and systemic diseases, known as protein conformational disorders (Soto and Pritzkow, 2018; Salahuddin et al., 2016). These disorders encompass common neurodegenerative diseases and rare inherited disorders characterized by the deposition of protein aggregates in the brain. Neurodegenerative diseases are characterized by a spectrum of conditions affecting cognitive functions, motor skills, emotions, memory, and other abilities (Christidi et al., 2018). This diverse group of diseases includes Alzheimer’s disease (AD), Parkinson’s disease (PD), Huntington’s disease (HD), as well as related polyglutamine disorders such as various forms of spinocerebellar ataxia (SCA), transmissible spongiform encephalopathies (TSEs), Ataxia-telangiectasia (A-T), and amyotrophic lateral sclerosis (ALS) (Marcos-Rabal et al., 2021). Extensive evidence from neuropathological and genetic studies, along with the generation of transgenic animal models, strongly supports the notion that diverse neurodegenerative diseases stem from the misfolding, aggregation, and accumulation of specific proteins in the brain (Soto and Pritzkow, 2018) resulting in high-order aggregate formation, imposing significant stress on neurons, and triggering cytotoxic events. These events include increased production of reactive oxygen species (ROS), excitotoxicity, synaptic neurodegenerative diseases in degradation systems, endoplasmic reticulum (ER) stress, DNA damage, mitochondrial dysfunction, inflammation, and cell cycle re-entry (Karvandi et al., 2023). Mishandling these challenges eventually culminates in neuronal death and activates the immune responses through microglial cells.

### 1.1 Chronic neuroinflammation and protein aggregation: insights from different model systems

The immune system is crucial for maintaining tissue homeostasis, eliminating pathogens, and aiding in injury recovery. Typically, immune responses are beneficial and self-limiting, resolving once tissue repair is complete or an infection is eradicated (Hill-Burns and Clark, 2009). However, if an inflammatory stimulus is not effectively cleared, the usual resolution mechanisms can become overwhelmed. Sustained chronic inflammation leading to the release of neurotoxic factors and worsening disease has been reported for many neurological disorders (Frakes et al., 2014; Xiang et al., 2023).

Neuroinflammation serves as an initial defense mechanism to safeguard the brain by eliminating or suppressing various pathogens (Morales et al., 2014; Kempuraj et al., 2017). This inflammatory reaction can offer beneficial outcomes by facilitating tissue repair and clearing cellular debris. However, prolonged inflammatory responses can be harmful as they hinder regeneration. The persistence of inflammatory stimulation may arise from endogenous factors such as genetic mutations and protein aggregation, or external factors like infections, trauma, and exposure to certain drugs. These sustained inflammatory responses involve the activation of microglia and astrocytes and can contribute to the development of neurodegenerative diseases (Xu et al., 2016; Kwon and Koh, 2020).

**FIGURE 1:**
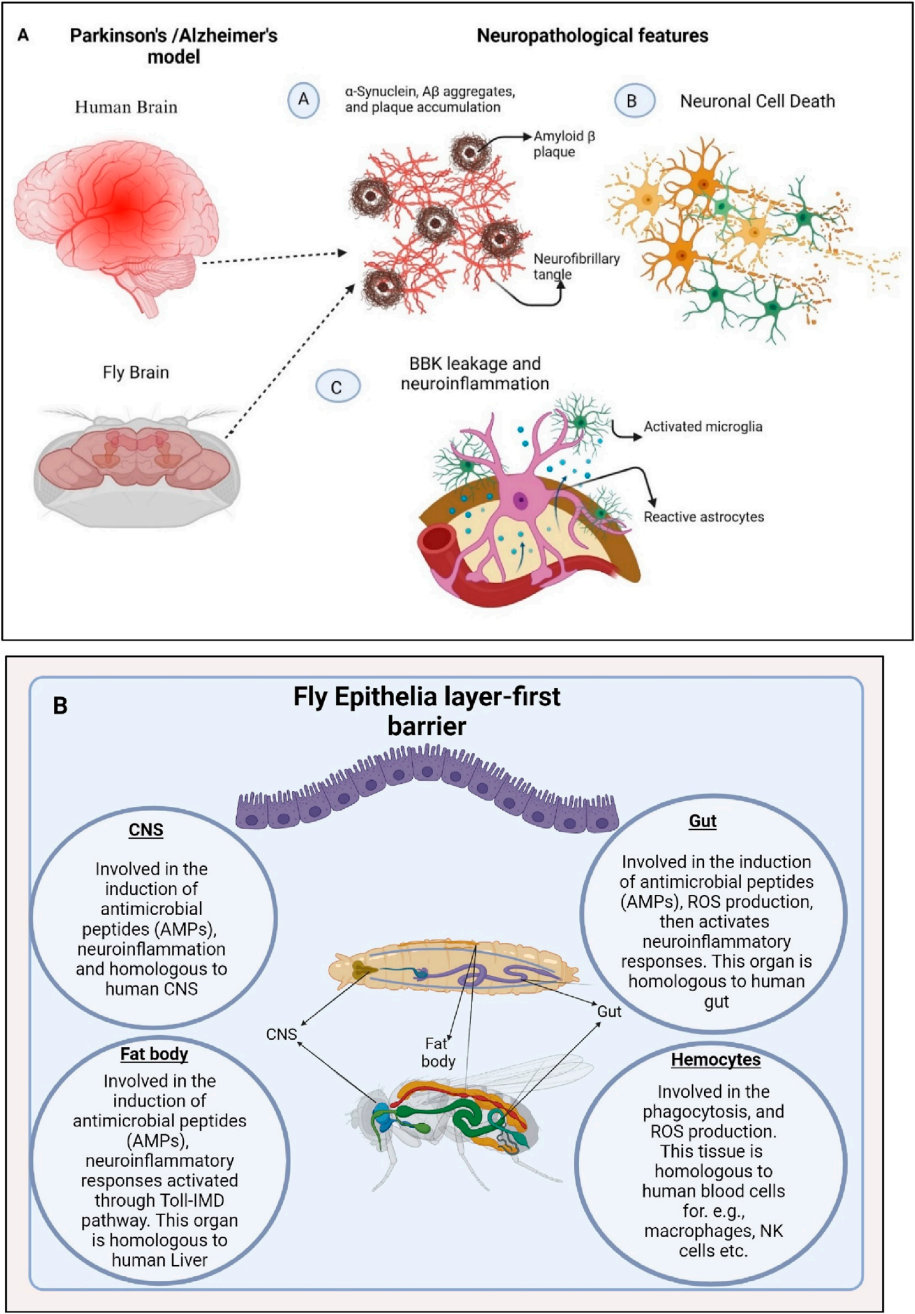
Neuronal cell death and enhanced production of antimicrobial peptides (AMPs) triggers neuroinflammation in *D. melanogast*er **(A)**. Misfolding of α-Synuclein and Aβ proteins causes neurotoxicity which further activates the immune responses via activation of microglial cells causing BBB (Blood Brain Barrier) leakage and neuroinflammation. **(B)**. The organs of the fly, which are crucial in activating molecules and signaling in the innate immune response, have striking functional similarities with those of mammals. The fly’s CNS, gut, trachea, fat body, Malpighian tubules, and hemocytes mimic their human counterparts and actively participate in triggering immune pathways, inducing the production of antimicrobial peptides (AMPs) and reactive oxygen species (ROS), melanization, cytokine production, and phagocytosis. Generated by BioRender.

Conformational disorders are characterized by the ability of specific proteins to fold into stable alternative conformations, leading to their aggregation and accumulation as fibrillar deposits within tissues of different model organisms as reported for mice and *D. melanogaster* (*Drosophila melanogaster*) (Iijima et al., 2008) (Figure 1A). The innate immune system is triggered by various factors such as microbial infiltration, injury, stress, aging, and brain disorders (Leszek et al., 2016). Excessive activation of the innate immune system and subsequent neuroinflammatory reactions contribute to chronic age-related neurodegeneration. Interestingly, the mechanism for immune pathway activation is similar between *D. melanogaster* and humans (Leclerc and Reichhart, 2004). Therefore, *D. melanogaster* can serve as a valuable model organism to examine and understand the cellular and molecular mechanisms underlying the connection between infection and neurodegenerative diseases.

**FIGURE 2:**
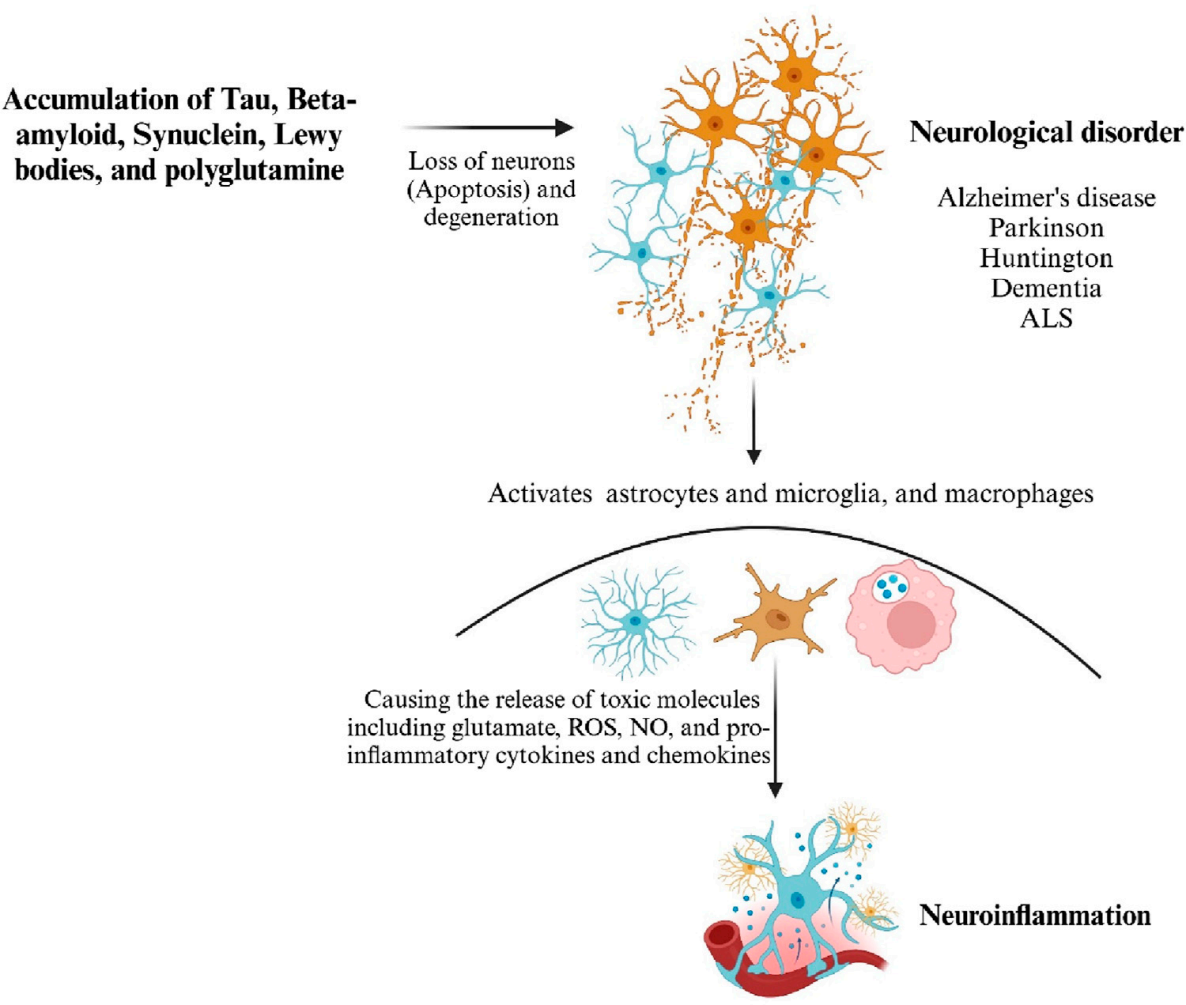
Misfolded proteins activate the innate immune response. Microglia, astrocytes and sustained chronic inflammation cause neurotoxicity and severe neuronal death. Generated by BioRender.

In *D. melanogaster*, prolonged and excessive neuroinflammatory responses in the brain can lead to neurodegeneration, facilitated by the neurotoxic effects of antimicrobial peptides (AMPs) (Dhankhar et al., 2020; Nayak and Mishra, 2022). In mammals, prolonged inflammation in microglial cells contributes to the progression of neurodegenerative diseases. While *D. melanogaster* does not possess specific microglial cells, they have different types of glia cells that can perform similar functions to microglia. As a result, the link between inflammatory processes in the brain and the pathogenesis of neurodegeneration in *D. melanogaster* has been systematically investigated and reported. Recent findings indicate a potential common cause and pathological mechanism underlying these diseases: the misfolding, aggregation, and accumulation of proteins leading to neuronal apoptosis followed by the induction of neuroinflammatory responses (Erekat, 2022; Nayak and Mishra, 2022) (Figures 1B, 2).

While these protein deposits share some morphological, structural, and staining characteristics, different protein aggregates may also exhibit distinct biochemical features, particularly depending on whether they accumulate intra- or extracellularly. The term “amyloid” was initially used to describe extracellular protein deposits found in AD and systemic amyloid disorders, but it has since been expanded to include certain intracellular aggregates. The first indications of involvement of protein misfolding and aggregation in neurodegenerative diseases emerged from post-mortem neuropathological studies (Chiti and Dobson, 2017). More than a century ago, Alois Alzheimer identified the characteristic neuropathological features of the disease bearing his name: neuritic amyloid plaques and neurofibrillary tangles (Alzheimer, 1907; Graeber et al., 1998; Möller and Graeber, 1998). Amyloid plaques, primarily composed of the amyloid-β protein (Aβ), accumulate extracellularly in the brain parenchyma and around cerebral vessel walls. Neurofibrillary tangles, on the other hand, consist of aggregates of hyperphosphorylated tau protein within the cytoplasm of degenerating neurons.

### 1.2 Genetic factors and protein aggregation in neurodegenerative diseases: the role of α-synuclein and other pathogenic proteins

Building on the understanding of neuroinflammation and protein aggregation, the genetic underpinnings of neurodegenerative diseases provide further insight into their pathogenesis. Notably, the observation that Parkinson’s disease (PD) can be transmitted in some families as either an autosomal dominant or an autosomal recessive trait indicates that mutations in single genes can cause certain forms of PD in a monogenic manner (Matsumine et al., 1997). The first gene associated with PD to be discovered was alpha (α)-Synuclein. It encodes a 143-amino acid, neuron-specific protein identified through expression screening from the electric organ of the fish *Torpedo californica* (Maroteaux et al., 1988). It was named “Synuclein” because the protein was found in both synapses and the nuclear envelope. α-Synuclein have been shown to be highly conserved across distantly related species (Jakes et al., 1994). In PD, neurons within the substantia nigra (SN) harbor cytoplasmic aggregates known as Lewy bodies, primarily comprised of fragments of α-Synuclein protein (Stefanis, 2012; Choong and Mochizuki, 2022). Meanwhile, intranuclear deposits of a polyglutamine-rich version of huntingtin protein are characteristic of HD. In ALS, aggregates containing superoxide dismutase (SOD1) are found in the cell bodies and axons of motor neurons (Sherman and Goldberg, 2001; Soto and Martin, 2009). Additionally, various forms of transmissible spongiform encephalopathies (TSEs) are characterized by the accumulation of protease-resistant aggregates of the prion protein (PrP) in the brains of affected humans and animals, sometimes resembling the amyloid plaques seen in AD.

Given these clinical observations, understanding the triggers of pathological changes leading to cell death and regional brain shrinkage is of paramount importance. This knowledge can ideally facilitate the development of treatments to counteract disease progression. Mature CNS neurons exhibit high resistance to cell death compared to immature neurons, owing to their ability to maintain cellular homeostasis and withstand various stresses. However, in neurodegenerative diseases, multiple stresses accumulate beyond the cell’s recovery capacity, leading to neuronal demise. Traumatic incidents such as ischemic strokes or prolonged seizures can also induce acute neuronal cell death by abruptly disrupting energy production in affected neurons (Chauhan, 2014; Kaur and Sharma, 2017).

Recent findings indicate that inflammation plays a causal role in the pathogenesis of various diseases, particularly late-onset CNS disorders. Understanding and managing the intricate crosstalk between the immune system and the nervous system could hold the key to preventing or delaying these conditions. Alzheimer’s disease demonstrates that its pathogenesis extends beyond neuronal processes and involves significant interactions with immunological mechanisms in the brain.

When misfolded and aggregated proteins bind to pattern recognition receptors on microglia and astroglia, they initiate an innate immune response marked by the release of inflammatory mediators. This response contributes significantly to the progression and severity of the disease (Béraud and Maguire-Zeiss, 2012). Upon receptor ligation, microglia begin to engulf Aβ fibrils through phagocytosis, leading these fibrils into the end lysosomal pathway. Unlike fibrillar Aβ, which is resistant to enzymatic degradation, soluble Aβ can be broken down by various extracellular proteases. Among these, neprilysin and insulin-degrading enzyme (IDE) play crucial roles within microglia. However, increased cytokine concentrations, resulting from the downregulation of Aβ phagocytosis receptors, may lead to inadequate microglial phagocytic capacity and subsequent neuronal apoptosis.

In *D. melanogaster*, dysregulation of different processes including autophagy, cell death, apicobasal polarity, sensory neuronal cell fate specification and maintenance, dendritic arborization establishment and maintenance, and optic lobe neuroepithelial differentiation leads to progressive neurodegeneration (Sahu and Mondal, 2020).

This review highlights the critical role of neuroinflammation in the CNS, emphasizing its impact on neuronal function and survival. The study focuses on microglia’s dual roles in neuroprotection and neurotoxicity, particularly in the context of PD and AD, using insights from *D. melanogaster*. By examining the regulatory mechanisms of AMPs and the contribution of various glial cell types, this review aims to advance understanding of neuroinflammation’s role in neurodegenerative disease progression, offering potential pathways for novel diagnostic and therapeutic strategies.

## 2 Common cell death mechanisms among neurodegenerative diseases

### 2.1 Autophagy and cell death

Autophagy is a finely regulated process essential for maintaining cellular homeostasis and responding to various cellular stresses. Many neurodegenerative diseases are marked by thebuild up of misfolded proteins and the gradual loss of specific neuronal cell populations. This key intracellular process, responsible for clearing aggregated proteins and damaged organelles, is increasingly acknowledged for its role in the development of pathological changes observed in conditions like AD, PD, HD and ALS. Dysregulated autophagy is believed to play pivotal roles in the progression of most neurodegenerative disorders, prompting exploration into autophagy regulation as a potential therapeutic strategy (Wu et al., 2018; Rai and Roy, 2022; Rai et al., 2023).

Autophagy encompasses three distinct subtypes: macroautophagy, microautophagy, and chaperone-mediated autophagy (Galluzzi and Green, 2019).While these subtypes vary in cargo recognition and the involvement of molecular chaperones, they converge at the lysosome for cargo digestion and recycling. The process of autophagy is often depicted as autophagic flux, comprising autophagosome formation, fusion with lysosomes, and cargo degradation within lysosomes. Initially, misfolded proteins and damaged organelles are sequestered by a newly formed membrane structure known as the phagophore. This membrane originates from various cellular sources, including the plasma membrane, Golgi, mitochondria, or ER. The phagophore gradually engulfs cargoes through elongation until it closes to forming an autophagosome. Subsequently, the autophagosome is transported to the lysosome via cytoskeletal microtubule systems, where it fuses to form an autolysosome. Within the autolysosome, lysosomal enzymes digest the cargoes, which are then recycled for reuse (Yamano et al., 2018).

Autophagy is orchestrated by numerous proteins, including several autophagy-related proteins found in mammals. Initiation of autophagy involves two major complexes: the ULK complex and the class III phosphatidylinositol-3-kinase (PI3K) complex, which are recruited to the phagophore assembly site (PAS). The ULK complex comprises ULK1/2, FIP200, and ATG13, while the PI3K complex, also known as the Beclin1 complex, consists of Vps34, p15, Beclin1, and Barkor (Guo et al., 2018). Notably, Beclin1, localized on the ER membrane, is regulated by anti-apoptotic dimers such as BCL-2 and BCL-XL. Upon autophagy activation, Beclin1 dissociates from the BCL-2 complex and works with Vps34, leading to the concentration of phosphatidylinositol 3-phosphate [PI (3)P] on the phagophore’s surface (R Kang et al., 2011). The extension and closure of the autophagosome are mediated by two ubiquitin-like complexes: the Atg5-Atg12-Atg16 complex and the LC3 (Atg8 ortholog) conjugation systems. Atg9, along with Atg2 and Atg18, facilitates trafficking between the Trans-Golgi network, endosomes, and newly formed autophagosomes (Kang et al., 2011) (Lystad et al., 2019). Following maturation, autophagosomes require kinesin and motor proteins to move along microtubules. Upon fusion with lysosomes, multiple membrane protein complexes, such as soluble NSF attachment protein receptors (SNAREs), are recruited. Once autolysosome formation is complete, the cargoes carried by autophagosomes undergo degradation by proteolysis (Lystad et al., 2019).

Macroautophagy, commonly referred to as autophagy, is a cellular process crucial for the removal of long-lived proteins and dysfunctional organelles that are too large for degradation by the proteasome. Autophagy not only contributes to fundamental cellular processes like development, differentiation, apoptosis, response to pathogen invasion, and adaptation to starvation, but it also impacts diseases ranging from cancer and immune disorders to neurodegenerative conditions. Numerous studies have established a close association between autophagy and neurodegenerative diseases. For instance, observations reveal significantly increased autophagic vacuoles in the brains of individuals with neurodegenerative diseases compared to healthy controls, indicating impaired maturation of autophagosomes into autolysosomes (Rai and Roy, 2022). Autophagy plays a crucial role in eliminating aggregated proteins implicated in various neurodegenerative diseases, such as mutant α-synuclein in PD, mutant huntingtin in HD, and mutant TAR DNA-binding protein 43 (TDP-43) in ALS (Furlong et al., 2000; Ojaimi et al., 2022). Inhibition of autophagy hampers the clearance of these toxic proteins, while their activation enhances their removal. Dysfunction of lysosomes in neurons is closely linked to neurodegeneration and cell death mechanisms. Accumulating genetic and biochemical evidence points to disruptions in endosomal–lysosomal and autophagic lysosomal pathways as contributors to the pathogenesis of many neurodegenerative diseases, including AD, PD, and ALS. The therapeutic potential of modulating autophagy/lysosomes in animal models further emphasizes the importance of lysosomal dysfunction in the pathogenesis of neurodegenerative diseases (Wolfe et al., 2013; Bordi et al., 2016).

The vulnerability of the brain in lysosomal disorders suggests that neurons may depend even more heavily on autophagy compared to other cell types for maintaining protein balance. Neurons face challenges due to their unique structures, such as extensive dendritic and axonal cytoplasm, making it difficult to efficiently remove damaged organelles and waste materials. With aging, neurons gradually lose their ability to effectively clear these waste products, leading to the accumulation of abnormal autophagic substrates. Consequently, neurons are particularly susceptible to damage caused by impaired autophagy and proteolysis.

Defects in the autophagy machinery in neuronal cells lead to cell death and neurodegeneration, which subsequently induces an inflammatory response causing neuroinflammation. This is a common pathophysiological mechanism in AD, PD, and ALS (Ramanan and Saykin, 2013).

### 2.2 The interrelation of neuroinflammation and neurodegeneration

Neurodegenerative diseases are characterized by the gradual loss of specific neuronal subsets, yet recent evidence suggests that immune responses also play a crucial role in disease progression. Host-pathogen interactions indicate a conserved innate immune function across species. In vertebrates, the presence of adaptive immune responses often overshadows the innate immune response, making vertebrate models less ideal for studying innate immunity. In contrast, *D*. *melanogaster* lacks adaptive immunity (Nayak and Mishra, 2022) and with similarities in neural development mechanisms and innate immune activation between flies and humans, coupled with advanced genetic tools, *D*. *melanogaster* emerges as an excellent model organism for studying immune responses in neurodegenerative diseases (Buchon et al., 2014).

Given the substantial homology between the human and *D. melanogaster* immune systems and recent findings highlighting the interplay between the immune system and neurodegenerative disease progression, this review aims to provide a comprehensive understanding of how neuro-immune interactions contribute to neurodegeneration, utilizing *D. melanogaster* as a model system.

The innate immune response of *D. melanogaster* to microbial infections is multifaceted, involving both humoral and cell-mediated reactions. The humoral response entails the production of AMPs from the fat body, which functions similarly to the mammalian liver. Within 24 h of microbial infection, the fat body releases AMPs into the hemolymph, reaching concentrations of up to 300 μM (Bulet and Stocklin, 2005; Lemaitre and Hoffmann, 2007). Additionally, *D. melanogaster’s* cellular immune response comprises three major types of surveillance cells, or hemocytes: plasmatocytes, lamellocytes, and crystal cells. Plasmatocytes, akin to mammalian macrophages, make up approximately 95% of circulating hemocytes and serve as professional phagocytes, also participating in AMP production (Lebestky et al., 2000). Like macrophages, plasmatocytes differentiate into tissue-resident cells, further solidifying their analogy with mammalian macrophages (Elrod-Erickson et al., 2000). Crystal cells, constituting the remaining 5% of circulating hemocytes, secrete components of the phenoloxidase cascade essential for pathogen melanization and wound healing. Lamellocytes, the largest and least abundant hemocytes in healthy larvae, are involved in encapsulating large invading pathogens such as wasp eggs (Lamberty et al., 2001; Meister and Lagueux, 2003). Mammals lack equivalent counterparts for crystal cells and lamellocytes.

Like in mammals, hematopoiesis in *D. melanogaster* also relies on the Janus kinase/signal transducers and activators of transcription (JAK/STAT) pathway and Notch signaling. Constitutive activation of the JAK/STAT pathway in humans can lead to leukemia, lymphoma, and developmental defects, mirroring the leukemia-like phenotype induced by hyper-activation of its homolog, Hopscotch, in *D. melanogaster* larvae (Luo et al., 2002).

**FIGURE 3:**
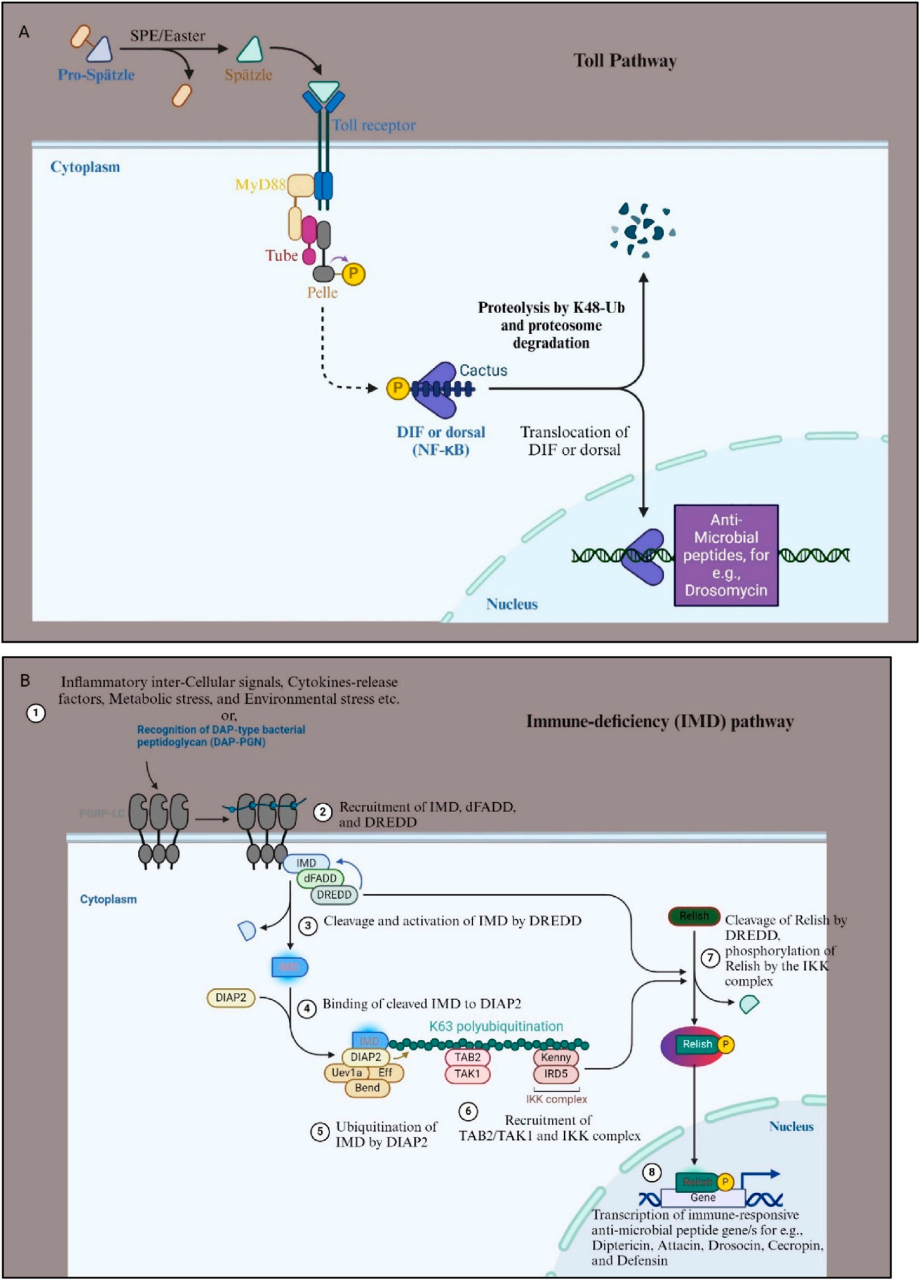
Toll and IMD signaling pathway of *D*. *melanogaster.* **(A)** The Toll pathway in *Drosophila melanogaster* is a critical component of the innate immune response, primarily activated by fungal and bacterial infections. It begins with the recognition of pathogens by pattern recognition receptors (PRRs), which detect microbial patterns. This leads to proteolytic activation of the cytokine-like molecule Spätzle. Activated Spätzle binds to the Toll receptor, which recruits adaptor proteins such as MyD88, Tube, and Pelle, initiating a phosphorylation cascade that results in the degradation of the Cactus (IkB homolog) protein. This degradation releases the transcription factors Dl and DIF, allowing them to translocate into the nucleus. Once in the nucleus, Dl and DIF induce the expression of AMPs like Drosomycin and other immune response genes, helping the organism to combat the infection. **(B)** The IMD pathway is primarily activated by aggregated proteins or Gram-negative bacterial infections. This pathway is initiated by the binding of bacterial peptidoglycans to the pattern recognition receptor PGRP-LC (step 1). Upon recognition, PGRP-LC activates the IMD protein, which recruits the adaptor protein death domain-containing protein (dFADD) and the caspase “death related ced-3/Nedd 2- like” (Dredd) (step 2). This leads to the activation of the Transforming growth factor-β (TGF-β)-activated kinase 1 (TAK1) complex, which subsequently activates the inhibitor of nuclear factor kappaB-kinase (IKK) complex (step 4, 5, and 6). The IKK complex phosphorylates the NF-κB-like transcription factor Relish, causing its cleavage (step 7). The cleaved Relish translocates to the nucleus, where it activates the transcription of AMPs for e.g., Attacin, Cecropin, Drosocin etc., and other immune response genes (step 8). Both pathways are essential for the *Drosophila melanogaster* immune defense, each tailored to respond to different types of pathogens. For more details see Kleino and Silverman, 2014. Figure drawn with Biorender.

The production of AMPs and cytokines in response to infection is governed by two distinct nuclear factor-kappa B (NF-κB) signaling pathways: Toll and immune deficiency (Imd) (Hoffmann and Reichhart, 2002) (Figure 3). These pathways, activated upon gram-positive bacterial and fungal infections (Toll pathway) or gram-negative bacterial infections (Imd pathway), respectively, regulate the differential expression of AMP-encoding genes through distinct NF-κB-like transcription factors (Figure 3) (Hoffmann and Reichhart, 2002; Martinelli and Reichhart, 2005). The Toll pathway is activated by an extracellular serine protease cascade, leading to the release of processed spätzle, analogous to mammalian Interleukin-17 (IL-17), which binds to the Toll receptor and initiates downstream signaling cascades culminating in the nuclear translocation of the NF-κB-like transcription factors Dorsal (Dl) and Dorsal-related immunity factor (Dif) (Figure 3A). In contrast, the Imd pathway relies on the peptidoglycan recognition receptor-LC (PGRC-LC) and results in the phosphorylation and cleavage of the NF-κB-like transcription factor Relish, leading to AMP gene expression (Figure 3B). While adaptive immune responses were traditionally believed to be restricted to mammals, the presence of a primitive form of adaptive immunity in *D. melanogaster* alongside its innate immune defenses has also been reported (Siffrin et al., 2007).

AMPs are crucial elements of the innate immune system across a wide range of species, including humans, animals, and plants, acting as the first line of defense against foreign invaders. The production of cathelicidin and defensin peptides has been recognized as a crucial component of human innate immunity. Some examples are provided in Table 1 (Rathinakumar and Wimley, 2010; Starr et al., 2018).

**TABLE 1:** Antimicrobial peptides (AMPs) produced under disease conditions in various organisms.

AMPs	Organism/s
α-defensin 1	*Homo sapiens*
α-defensin 6	*Homo sapiens*
β-defensin 1	*Homo sapiens*
Antifungal heliomicin	*Heliothis virescens*
Defensin-like peptide-2	*Ornithorhynchus anatinus*
Fowlicidin-1	Chicken
LL-37 (hCLD)	*Homo sapiens*
Protegrin PG-5	*Sus scrofa*
Defb1 (Homolog to human β-defensin)	Mouse

### 2.3 Apoptosis of dopaminergic neurons activates neuroinflammatory responses in PD pathology

The innate immune system is primarily activated by microbial infiltration, injury, stress, aging, and brain disorders. Hyperactivation of this system and subsequent neuroinflammatory reactions contribute to chronic age-related neurodegeneration. The mechanisms for activating the immune pathway are conserved between *D*. *melanogaster* and humans (Nayak and Mishra, 2022).

PD is characterized by distinctive movement disorder symptoms such as tremors and postural instability, as well as specific pathological features including the accumulation of α-Synuclein and the loss of dopaminergic neurons in the substantia nigra (SN). α-Synuclein aggregates form Lewy bodies, which are found in the brains of PD patients (Erekat, 2018; Rai et al., 2023). Neuronal death in PD is largely attributed to apoptosis, a process involving various caspases that can be initiated through intrinsic or extrinsic pathways. The intrinsic pathway is mediated by caspase-9 activation, whereas the extrinsic pathway involves caspase-8 activation. Both pathways converge on executioner caspases like caspase-3 and caspase-7, leading to apoptosis characterized by DNA cleavage and fragmentation. Mitochondrial dysfunction is an early event in PD, observed both in humans and animal models (Lin and Beal, 2006). The SN in PD patients exhibit defects in mitochondrial complex I activity. Dopamine metabolism generates reactive oxygen species (ROS), which can trigger apoptotic cell death. Monoamine oxidase (MAO) metabolizes dopamine to produce H_2_O_2_, leading to the generation of ROS. Dopamine degradation products further contribute to increased ROS production (Rai and Roy, 2022). Dopaminergic neurons, susceptible to mitochondrial complex I dysfunction, are particularly affected, potentially leading to apoptosis.

Inherited forms of PD involve mutations in genes related to mitochondrial health, such as the E3-ubiquitin ligase *Parkin,* Leucine-rich repeat kinase 2 (*LRRK2),* PTEN-induced Kinase 1 *(PINK1),* and the protein deglycase *DJ-1* (*PARK7*)*.* These mutations underscore the vulnerability of nigral neurons to mitochondrial damage and dysfunction. Parkin deficiency, for instance, leads to mitochondrial dysfunction and contributes to PD pathology. Parkin plays multiple roles including promoting mitochondrial biogenesis and degrading toxic protein aggregates. *PINK1* mutations also link to mitochondrial dysfunction, where *Parkin* can compensate for *PINK1* mutations. *DJ1* mutations increase oxidative stress and disrupt mitochondrial function, contributing to early onset PD. *LRRK2* mutations induce defective mitochondrial dynamics and increase ROS production, potentially leading to dopaminergic neuronal death via apoptosis. Neuroinflammation also plays a significant role in PD pathogenesis, alongside mitochondrial dysfunction and impaired proteostasis. Chronic inflammation, triggered by risk factors such as α-Synuclein misfolding, immune-related gene polymorphisms, and mitochondrial dysfunction, initiates neurodegeneration. This neurodegeneration, in turn, induces immune responses, with microglia, astrocytes, and peripheral circulating myeloid cells actively participating in the neuroinflammatory process. This ongoing neuroinflammation further exacerbates PD pathology.

#### 2.3.1 PD and neuroinflammatory responses in *Drosophila melanogaster*

Stress stemming from intracellular misfolded proteins is associated with heightened immune responses, indicating the significant involvement of both innate and adaptive immune systems in neurodegeneration (Olejniczak et al., 2015). Unlike mammals, *D. melanogaster* depends solely on innate immunity, which comprises cellular and humoral components, to combat infections. In *D. melanogaster*, the Toll and IMD pathways orchestrate humoral immunity, prompting the production of AMPs to fend off bacterial and fungal infections. However, prolonged activation of individual AMPs has been linked to neurotoxic effects and the upregulation of caspases, eventually leading to significant neuronal apoptosis (Petersen et al., 2013) (Dulovic et al., 2014).

Neuroinflammation has been shown to contribute to neuronal death in diseases such as PD and AD (Giridharan et al., 2019; Kempuraj et al., 2019; Wu et al., 2019). For example, in AD, the accumulation of β-amyloid aggregates leads to persistent immune system activation and hinders microglial clearance.

In ataxia-telangiectasia model in *D. melanogaster*, Peterson et al. demonstrated that mutations in the protein kinase ataxia telangiectasia mutated (*ATM*) gene trigger nuclear translocation and activation of Relish, a key transcription factor activated by the IMD signaling pathway (Petersen et al., 2013). This nuclear translocation triggers the transcription and synthesis of AMPs such as Diptericin B (DptB) which plays a neuroprotective role at the initial level. Earlier studies also reported that during neurodegeneration, autoinflammation occurs, a condition known as “inflammaging.” Inflammation protects the host from microbial infection and stress-injury by activating microglia and astrocytes in the CNS. However, chronic inflammation arises due to the prolonged activation of microglial and macrophages constantly producing AMPs, ROS and cytokines which further promote damage to neurons (mainly motor neurons, and dopaminergic) which culminate in development of PD.

In mammalian models, NF-κB is crucial for the inflammatory response by regulating genes that encode pro-inflammatory mediators. Elevated NF-κB activation has been observed in the brains of PD patients, highlighting the connection between immune activation and neurodegenerative diseases. Similarly, in *D. melanogaster* increased NF-κB activation has been associated with age-related neurodegeneration. Thus, precise regulation of the IMD pathway is essential to prevent uncontrolled chronic inflammation (Maitra et al., 2019).

Multiple genes associated with PD have been identified, significantly advancing our understanding of its etiology and treatment (Larsen et al., 2018). The death of dopaminergic neurons in PD is accompanied by astrocytic dysfunction, hyper-activation of microglia, and the activation of various inflammatory networks in the SN. α-Synuclein, the main component of Lewy bodies, forms abnormal aggregates not only in neurons but also in microglia. There is ongoing debate about whether α-Synuclein can trigger microglial responses and pathological reactions. Studies have shown that α-Synuclein disrupts neuronal function and activates microglia, leading to increased phagocytic activity and the production of pro-inflammatory cytokines (Lim et al., 2018).

**FIGURE 4:**
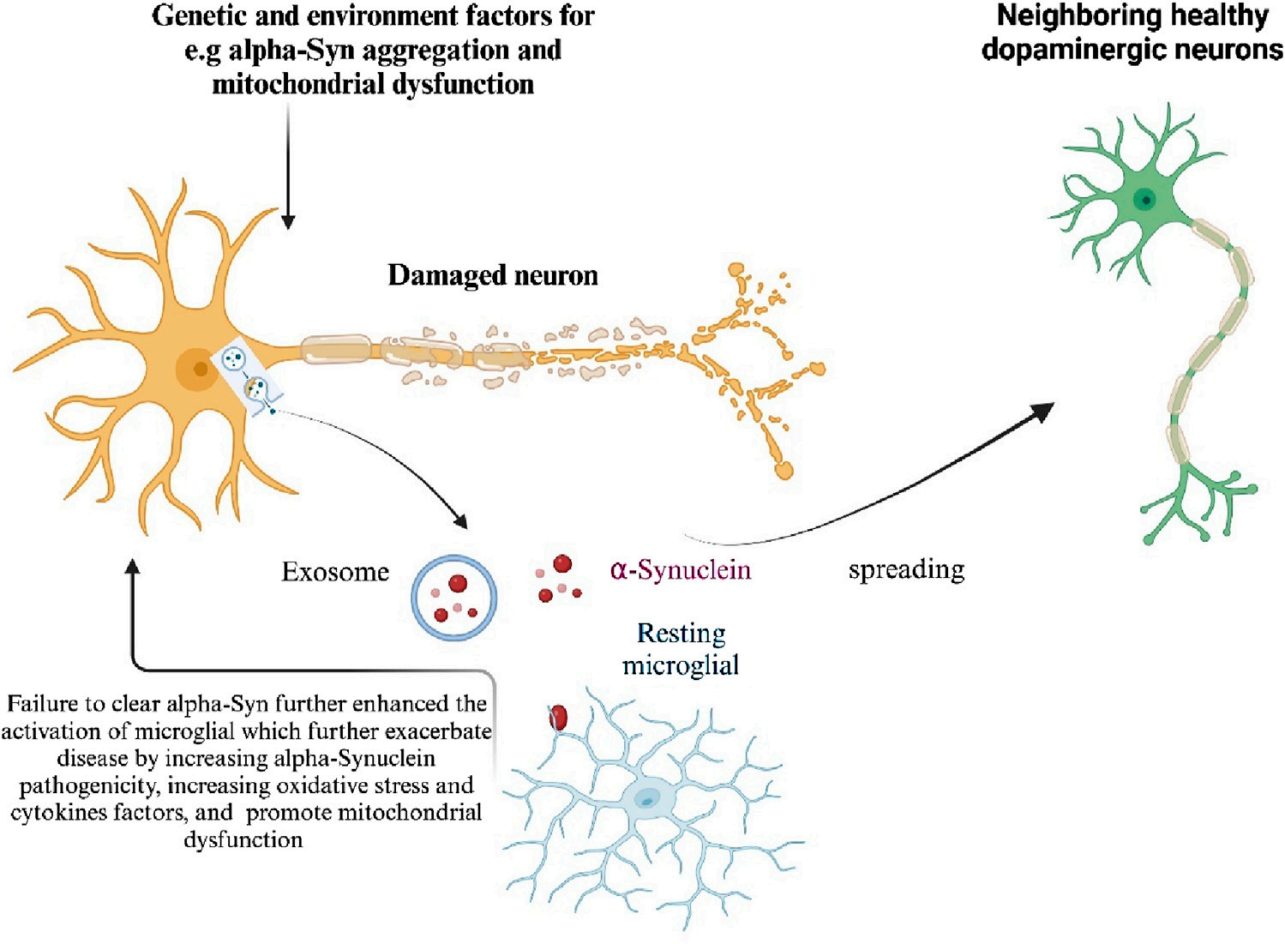
Mechanisms of α-Synuclein induced neurotoxicity in Parkinson’s Disease Genetic and environmental factors contribute to the aggregation of α-Synuclein, leading to its accumulation in neurons. Excessive α-Synuclein is transported into mitochondria, causing mitochondrial dysfunction, a key factor in PD progression. Mutations in mitochondrial-associated proteins such as LRRK2, PINK1, PARK7, and PRKN, found in familial PD cases, also induce mitochondrial dysfunction and neurotoxicity. When α-Synuclein aggregation exceeds cellular clearance capacity, it is released either directly from neurons or through exosomes, activating microglia and spreading α-Synuclein to neighboring healthy dopaminergic (DA) neurons, thus amplifying neurotoxicity. Damage-associated molecular patterns (DAMPs) released from dying neurons further enhance microglial activation. Additionally, genetic and environmental factors promote the activation of microglia and other immune cells. Activated microglia exacerbate the disease by increasing α-Synuclein pathogenicity, oxidative stress, and mitochondrial dysfunction. Generated by BioRender.

Although α-Synuclein is typically a cytosolic protein, a small amount can be released from neurons via the exocytosis process. Misfolding and aggregation facilitate its release from neuronal cells. Once released, α-Synuclein can be transferred to neighboring neurons and astroglia, where it promotes the formation of inclusion bodies, induces cell death in neurons, and triggers proinflammatory responses from microglia and astroglia (Kim et al., 2013). Additionally, cell-released α-Synuclein acts as an endogenous agonist for Toll-like receptor 2 (TLR2), which activates microglia and leads to neurotoxicity (Figure 4). By eliminating the interaction between neuron-released α-Synuclein and TLR2, inflammatory responses in the brain are dampened, presenting a potential therapeutic approach (Kim et al., 2013). Overexpression of α-Synuclein *in vivo* also increases microglial activation (Drouin-Ouellet et al., 2015). However, α-Synuclein internalization proceeds even in the absence of TLR2, indicating that its uptake involves multiple receptor systems (Drouin-Ouellet et al., 2015). The transcription factor NF-κB regulates neuroinflammation in glial cells, contributing to the pathology of several neurodegenerative diseases, including PD (Shabab et al., 2017). NF-κB activation increases the production of pro-inflammatory cytokines, chemokines, inducible nitric oxide synthase (iNOS), and cyclooxygenase-2 (COX-2), leading to neuroinflammation (Ghosh and Hayden, 2008). Interestingly, NF-κB activation in neurons promotes survival and plasticity (Mettang et al., 2018), whereas in glial cells, it accelerates inflammatory processes in neurodegenerative diseases (Frakes et al., 2014; Bernaus et al., 2020).

In the α-Synuclein mutant PD model in *D. melanogaster*, a high level of Relish expression is observed in the larval CNS which indicates the activation of innate immune inflammatory responses, and the loss of dopaminergic neurons are also marked in the larval as well as adult brain, as evident of neuroinflammation effects. Physiological changes have also been observed like significant reduction in the climbing ability and eye-roughness (Rai et al., unpublished data).

In addition to this, mitochondrial dysfunction also plays an important role in neuroinflammation. The genes *PARK2* and *PINK1*, which cause autosomal recessive forms of PD, maintain mitochondrial homeostasis through mitochondrial homeostasis (Rai and Roy, 2022). Mitochondrial dysfunction is closely linked to PD, warranting further study of mitophagy dysfunction in PD pathogenesis (Rai et al., 2023; 2024). The NLRP3 inflammasome (NOD-, LRR- and pyrin domain-containing protein 3) is an indispensable component of the innate immune system that facilitates caspase-1 activation and the secretion of proinflammatory cytokines IL-1β/IL-18 in response to microbial infection and cellular damage. Depleting mtDNA and mtROS in p62ΔMye macrophages reduces excessive IL-1β production in response to NLRP3 agonist stimulation, highlighting the dependency on mitochondrial damage (Shi et al., 2015). In *in vitro* assays, mitochondrial lysates induce inflammation in primary microglia and increase the mRNA levels of cytokines like tumor necrosis factor (TNFα) and IL-8 (Wilkins et al., 2015). ROS from damaged mitochondria activate the NLRP3 inflammasome pathway and other redox-sensitive proteins (Heid et al., 2013).

Mitochondrial dysfunction releases various pro-inflammatory molecules, including mitochondrial DNA, ATP, cardiolipin, mitochondrial transcription factor A, cytochrome c, formyl peptides, and RNA (Sliter et al., 2018). Damage-associated molecular patterns (DAMPs) from mitochondria trigger immune responses and mitophagy (West, 2017). Inflammation-induced mitochondrial dysfunction and dopaminergic neurodegeneration can be mitigated by anti-inflammatory drugs (Hunter et al., 2007; Yang et al., 2020). Clinically, modulating the immune system is a promising strategy for treating PD, given the significant role of neuroinflammation in its progression (Kustrimovic et al., 2018). While previous studies suggested that neurodegenerative disease inflammation originates in the brain’s microglia, other views propose that it may also originate from peripheral circulation (Braak et al., 2003).

### 2.4 Clinical application

Many fundamental biological, physiological, and neurological properties are conserved between mammals and *D. melanogaster*, with nearly 75% of human disease-causing genes believed to have a functional homolog in the fly. Several models of human neurodegenerative diseases e.g., AD and PD have been developed using *D. melanogaster* and other models, demonstrating their potential for drug discovery (Table 2) (Jackson et al., 2002; Springer et al., 2005; Luheshi et al., 2007; Sang et al., 2007; Cao et al., 2010; Feuillette et al., 2010). These models share common phenotypic traits in flies, such as retinal degeneration, locomotor defects, wing abnormalities, climbing impairments, and reduced lifespan (Table 2). Thus, drug discovery assays aimed at identifying therapeutics for these neurodegenerative diseases can utilize protocols focused on these shared phenotypes. For instance, potential drugs could be evaluated based on their ability to rescue the rough eye phenotype, improve locomotor and climbing deficits, or restore normal activity levels.

**TABLE 2:** Disease-causing factors in AD and PD, and their role for neurodegeneration in various models.

Disease/Gene	Model	Phenotypes
Alzheimer’s disease		
β-Amyloid protein	*C. elegans*	Progressive paralysis, cytoplasmic protein accumulation, fibrillar amyloid formation
	*D. melanogaster*	Eye degeneration, amyloid plaque accumulation, reduced lifespan, locomotor defects, brain vacuolation
	*Mouse*	Eye degeneration, amyloid plaque accumulation, reduced lifespan, locomotor defects
Tau proteins		
	*C. elegans*	Age-dependent neurodegeneration, insoluble tau accumulation, reduced lifespan, progressive impairment in touch response, embryonic lethality, mechanosensory defects
	*D. melanogaster/Mouse*	Eye degeneration, disruption of microtubular networks at presynaptic nerve terminals, axonal degeneration, morphological defects at neuromuscular junctions
Parkinson’s Disease		
α-Synuclein	*C. elegans*	Mitochondrial stress, dopaminergic neuron degeneration, developmental defects, increased dopamine synthesis, redistribution of dopaminergic synaptic vesicles
	*D. melanogaster/Mouse*	Age-dependent dopaminergic neuron loss, progressive climbing defects
Parkin and Pink1		
	*C. elegans*	Hypersensitivity to proteotoxic stress, Parkin insolubility and aggregation
	*D. melanogaster/Mouse*	Dopaminergic neuron loss, age-dependent motor deficits, reduced lifespan, locomotor defects, male sterility, mitochondrial pathology

Therapeutic approaches targeting the peripheral immune system have also shown promise in preclinical trials (Choi et al., 2019). Adoptive transfer of T cells immunized with glipidine acetate to PD mice suppressed microglial activation and provided neuroprotective effects. The gut-brain α-Synuclein transmission model, proposed by Heiko Braak and colleagues based on post-mortem studies, has gained support from recent evidence (Braak et al., 2003). They proposed that misfolding of α-synuclein, triggered by an external factor in the enteric nervous system (ENS), neuropod cells, and the olfactory bulb, leads to the accumulation of Lewy bodies if clearance mechanisms are impaired. Subsequently, α-Synuclein aggregations migrate to the brainstem via the vagus nerve or through olfactory structures. Finally, the aggregations reach the especially susceptible dopaminergic neurons of the SN, resulting in disease symptoms. However, the modulation of immune activity is crucial for gut-brain communication, with chronic pro-inflammatory immune activity being a key element in neurodegenerative diseases. Intestinal inflammation is relevant to PD pathogenesis, necessitating further study of the inflammation mechanisms involved in gut-brain α-Synuclein transmission.

In conclusion, neuroinflammation, driven by the activation of microglia, astrogliosis, and other key pathways, plays a significant role in neurodegeneration. Pharmacological modulation of neuroinflammation may offer a therapeutic strategy for treating neurodegenerative diseases.

## 3 Conclusion

Neuroinflammation is a key aspect of the chronic innate immune response in the CNS, contributing to neuronal dysfunction and death. The infiltration of foreign invaders or neuronal injury activates pro-inflammatory molecules secreted by the host immune system, leading to the accumulation of microglial cells and deregulation of brain tissue homeostasis, which can escalate into neurotoxicity or neurodegeneration. Prolonged expression of pro-inflammatory cytokines or AMPs derived from glial cells in the CNS of *D*. *melanogaster* results in elevated deposition of endogenous non-infectious ligands, such as α-Synuclein, contributing to the pathogenesis of PD. While AMPs have both protective and pathological roles in the brain in *D. melanogaster*, the regulatory mechanisms behind this functional switch remain unclear. Activated microglia have been observed in areas of neuronal damage and degeneration in post-mortem brains of patients with PD and AD. Research has shown that inflammatory responses are functionally linked to disease progression, with microglia exerting neuroprotective effects and inducing neurogenesis mainly through the release of cytotoxic soluble factors.

Microglial cells, as key players in the brain’s innate immune response, assume crucial and complex roles in various neurodegenerative disorders. They can change their morphological and phenotypic activation states in response to their microenvironment and potent activators, such as cytokines and chemokines, which induce both pro- and anti-inflammatory states. While the function of microglial cells has been extensively studied, the roles of other glial cells, including astrocytes, remain less clear. The involvement of dysregulated inflammation complicates the deployment of therapeutic interventions that could slow or halt disease progression. Understanding these mechanisms using *D. melanogaster* will aid in the development of novel diagnostic tools and therapeutics for neurodegenerative diseases.

## Figures and Tables

**Table:** 

**AD**	Alzheimer’s Disease
**ALS**	Amyotrophic Lateral Sclerosis
**AMPs**	Antimicrobial Peptides
**ARM**	Armadillo repeats
**A-T**	Ataxia-telangiectasia
**ATM**	Ataxia Telangiectasia Mutated
**BBB**	Blood Brain Barrier
**CNS**	Central Nervous System
**COX 2**	Cyclooxygenase-2
**DAMPs**	Damage-Associated Molecular Patterns
**dFADD**	*Drosophila* Fas-associated protein with death domain
**Dif**	Dorsal-immunity related factor
**D**	melanogaster (*Drosophila melanogaster*)
**DNs**	Dopaminergic Neurons
**DptB**	Diptericin B
**ER**	Endoplasmic Reticulum
**Dredd**	death related ced-3/Nedd 2- like
**HD**	Huntington’s Disease
**IKK**	inhibitor of nuclear factor-kappaB kinase
**Imd**	Immune Deficiency
**iNOS**	Nitric Oxide Synthase
**IDE**	Insulin-degrading enzyme
**IL-17**	Interleukin-17
**JAK/STAT**	Janus kinase/signal transducers and activators of transcription
**LRRK2**	Leucine Rich Repeat Kinase 2
**MAO**	Monoamine Oxidase
**NF-κB**	Nuclear Factor-Kappa B
**NLRP3 inflammasome**	NOD-, LRR- and Pyrin Domain-containing Protein 3
**PAS**	Phagophore Assembly Site
**PI3K**	Phosphatidylinositol-3-kinase
**PD**	Parkinson’s Disease
**PGRC-LC**	Peptidoglycan Recognition Receptor-LC
**PI 3P**	Phosphatidylinositol 3-Phosphate
**PrP**	Prion protein
**PINK1**	PTEN-induced Kinase
**ROS**	Reactive Oxygen Species
**SCA**	Spinocerebellar Ataxia
**SN**	Substantia Nigra
**SNAREs**	Soluble NSF Attachment Protein Receptors
**SOD1**	Superoxide Dismutase 1
**TAK1**	Transforming growth factor-β (TGF-β)-activated kinase 1
**TDP 43**	TAR DNA-binding protein 43
**TLR**	Toll-like receptor
**TNFα**	Tumor Necrosis Factor
**TSEs**	Transmissible Spongiform Encephalopathies
